# Mining Adverse Drug Reactions from online healthcare forums using Hidden Markov Model

**DOI:** 10.1186/1472-6947-14-91

**Published:** 2014-10-23

**Authors:** Hariprasad Sampathkumar, Xue-wen Chen, Bo Luo

**Affiliations:** EECS, University of Kansas, 66045 Lawrence, USA; Dept. of Computer Science, Wayne State University, 48202 Detroit, USA

**Keywords:** Adverse drug reaction, Pharmacovigilance, Text mining, Machine learning, Online healthcare forums, Hidden Markov model

## Abstract

**Background:**

Adverse Drug Reactions are one of the leading causes of injury or death among patients undergoing medical treatments. Not all Adverse Drug Reactions are identified before a drug is made available in the market. Current post-marketing drug surveillance methods, which are based purely on voluntary spontaneous reports, are unable to provide the early indications necessary to prevent the occurrence of such injuries or fatalities. The objective of this research is to extract reports of adverse drug side-effects from messages in online healthcare forums and use them as early indicators to assist in post-marketing drug surveillance.

**Methods:**

We treat the task of extracting adverse side-effects of drugs from healthcare forum messages as a sequence labeling problem and present a Hidden Markov Model(HMM) based Text Mining system that can be used to classify a message as containing drug side-effect information and then extract the adverse side-effect mentions from it. A manually annotated dataset from http://www.medications.comis used in the training and validation of the HMM based Text Mining system.

**Results:**

A 10-fold cross-validation on the manually annotated dataset yielded on average an F-Score of 0.76 from the HMM Classifier, in comparison to 0.575 from the Baseline classifier. Without the Plain Text Filter component as a part of the Text Processing module, the F-Score of the HMM Classifier was reduced to 0.378 on average, while absence of the HTML Filter component was found to have no impact. Reducing the Drug names dictionary size by half, on average reduced the F-Score of the HMM Classifier to 0.359, while a similar reduction to the side-effects dictionary yielded an F-Score of 0.651 on average. Adverse side-effects mined from http://www.medications.comand http://www.steadyhealth.comwere found to match the Adverse Drug Reactions on the Drug Package Labels of several drugs. In addition, some novel adverse side-effects, which can be potential Adverse Drug Reactions, were also identified.

**Conclusions:**

The results from the HMM based Text Miner are encouraging to pursue further enhancements to this approach. The mined novel side-effects can act as early indicators for health authorities to help focus their efforts in post-marketing drug surveillance.

## Background

Pharmaceutical drugs or medicines are chemical substances prescribed for the prevention, treatment or cure of diseases and other health conditions. A *side-effect* is an unintended response or reaction that is experienced by a patient due to the consumption of a drug. Side-effects can be both positive or negative, however, it is the negative side-effects or *Adverse Drug Reactions* (ADRs) that are more important, as they can severely affect the health of patients, sometimes fatally. In the United States (US), it is estimated that over 2 million serious ADRs occur among hospitalized patients, which results in over 100,000 deaths each year [1, 2] making ADRs a significant public heath problem.

Drugs are approved for use by general public only if their therapeutic effect outweighs their adverse side-effects. Drug manufacturers are mandated to publish the side-effects that have been identified as a part of the clinical trials. These are usually published as a part of the Drug Package Inserts or Drug Package Labels for each drug. However, the clinical trials are often not extensively enough to uncover all possible side-effects due to the small number and diversity of the participants involved. In order to address this issue, health organizations around the world employ post-marketing surveillance programs as a part of their *Pharmacovigilance*: the science relating to the detection, assessment, understanding and prevention of adverse effects of pharmaceutical drugs.

In the US, the Food and Drug Administration (FDA) has a post-marketing drug surveillance program called *MedWatch*, to monitor the effects of drugs once they have been released to the general public. MedWatch allows spontaneous reporting of adverse drug reactions by both healthcare professionals and patients. All the reported adverse events are recorded as a part of the FDA Adverse Event Reporting System (FAERS) and are constantly monitored for statistically significant adverse drug event reports. Once such reports are confirmed against a drug, the FDA may take necessary action against the drug manufacturer, sometimes by completely recalling the drug from the market. However, with the spontaneous reports being purely voluntary, not all adverse events get reported. It could take several years before a significant number is reported to initiate inquiry, analysis and follow up action, during which, the drug could continue to affect a larger percentage of the general population. Thus there is a need for systems that can help in the early detection of such adverse drug events.

Methods for automatic extraction of adverse drug events can be categorized based on the nature of the data sources: *structured* and *unstructured*. The spontaneous adverse event reports collected by the health authorities are the major sources for structured data, which though varying in format, are suitable for data mining. Reviews on data mining algorithms that have been used to extract adverse side-effects of drugs from such structured data sources are discussed in [3–10]. Information on adverse reactions of drugs is also widely available as a part of unstructured data sources such as: literary sources like published biomedical literature including books, journals and papers, along with clinical sources like patient medical history and online healthcare forums.

### Biomedical sources

Biomedical sources include text available in books, scientific papers, journals, drug package labels and similar published scientific literature. Information available in such sources are almost always free from grammatical and spelling errors and often follow a standard terminology which makes it easier to apply standard Natural Language Processing (NLP) techniques to extract useful information. SIDER [11] is an example of a resource that was used to capture phenotypic effects of drugs which are extracted from Drug Package Inserts available from public sources. More recently, there have been efforts to mine adverse drug reactions from PubMed citations [12] and even from Letters to the Editor of the journals [13] in which the related papers where published. Though these literary sources contain the most accurate information on the side-effects of drugs, they usually do not contain the most up-to-date information.

### Clinical sources

Clinical sources include information collected in a clinical setting like a patient’s personal medical history, physician’s notes, lab reports and discharge summaries. The information available in such sources often tend to be narratives that may contain spelling and grammatical errors along with short hand notations and ambiguous abbreviations. Mining of such clinical data has been considered to be unique [14] due to the ethical, legal and social constraints in access to privacy-sensitive information of the patients. One of the earlier efforts to extract information from clinical text was by Jang et al. [15] who made use of a Hidden Markov Model based semantic tagger to identify symptoms, therapeutic methods and performance information in clinical documents containing a mixture of English and Korean words. In recent years, most of this information is available in electronic format as Electronic Health Records (EHRs) enabling easier processing of data. Meystre et al. [16] provide reviews of such methods. Wang et al. [17] present a feasibility study of using Natural Language Processing and Statistics on EHRs to support active computerized Pharmacovigilance, while Warrer et al. [18] review text mining techniques on electronic patient records to identify ADRs from medicine use. Several research efforts like [19], [20] and [21] have been undertaken to mine data from EHRs. More recently, Sohn et al. [22] used a rule-based method to extract physician-asserted drug side-effects from clinical narratives of psychiatry and psychology patients and Liu et al. [23] examined the use of retrospective medication orders and inpatient laboratory results documented in the EHRs to identify ADRs. In spite of several such efforts, privacy concerns and security restrictions to access patient health records prevent a large volume of this source from being used for mining novel information.

### Online healthcare forums

More recently the growth of online social networks and healthcare forums has led patients to voluntarily share information about their health, treatments and drug use. Medications.com [24], SteadyHealth.com [25], MedHelp.org [26] and HealthBoards.com [27] are examples of such online forums that have lowered the barrier for patients to report their experiences, thereby acting as valuable sources for collecting first hand adverse event information. It is the information available in such online healthcare forums that we hope to leverage in our efforts to assist the health authorities in their post-marketing drug surveillance. Unlike text from biomedical and clinical sources, text from online healthcare forums is of free form and suffers from ungrammatical, misspelled and ambiguous words, making it a challenge for extracting useful information. However, the potential for having unrestricted access to the latest and first-hand information from the patients has motivated several research efforts to explore the possibility of extracting adverse drug side-effects from such online forums.

Leaman et al. [28] were among the early researchers to extract adverse side-effects from online healthcare forums. They collected user comments from the DailyStrength.com forum to identify adverse effects of six drugs that act on the central nervous system. They created a lexicon of adverse effects and used a sliding window approach to find strings in the user comments that are similar to their lexicon and thereby identify adverse reactions. There have been several approaches since then to extract ADRs from online forum messages.

Li [29] applied statistical techniques on user messages collected from pharmaceutical drug review sites, to identify significant associations between the statin class of drugs and a wide range of disorders, which could be corroborated based on existing research literature. More recently, Wu et al. [30] proposed UDWarning, an early warning system for discovering unrecognized drug side-effects. They make use of co-occurrence statistics of related side-effects to compute the relevance of a web page containing a drug and a side-effect. An increase in volume of high relevance web pages with an unrecognized side-effect is used to generate a warning for a drug. Similarly, Liu et al. [31] propose AZDrugMiner a framework built on statistical learning to extract patient-reported adverse drug events from online patient forums.

Among the Natural Language Processing approaches, Chee et al. [32] performed sentiment analysis using an ensemble of classifiers to identify drugs that can potentially fall under the FDA’s Watchlist category using messages posted as a part of the Health & Wellness Yahoo! Groups. Bian et al. [33] analyze the content of twitter messages by using Natural Language Processing to extract both textual and semantic features based on concepts returned by the UMLS meta thesaurus and use Support Vector Machine(SVM) based classifiers to mine ADRs. Recently, Yates et al. [34] developed the ADRTrace system based on lexicons, pattern identification and a synonym set including variations of medical terms in order to identify ‘expected’ and ‘unexpected’ ADRs.

Nikfarjam et al. [35] extended the work done by Leaman et al. to use association rule mining for identifying patterns which were then used to predict ADRs. Similarly, Yang et al. [36] also used association mining and Proportional Reporting Ratios to extract the associations between drugs and adverse reactions from the user contributed content in social media. Karimi et al. [37] are currently working on using heuristics and rule based approaches to extract both adverse and beneficial side-effects of drugs from online patient forums, along with the background information of patients.

In addition to the above approaches that work directly on the information available in the healthcare forums, there are also approaches like [38] and [39] that make use of the information from the search logs of such forums to extract adverse drug reactions.

As another alternative to the methods described above, we treat the task of extracting the adverse drug side-effect information from forum messages as a sequence labeling problem and propose a Hidden Markov Model (HMM) based Text Mining system to accomplish this. We believe the messages posted in the healthcare forums tend to follow a sequence of cause and effect when describing an association between a drug and its side-effect and model this association using the state sequences of a Hidden Markov Model. The proposed HMM based Text Miner on average yielded an F-Score of 0.76 across multiple runs of a 10-fold cross-validation on the manually annotated data set. The adverse side-effect information mined from the unseen messages of http://www.medications.com and http://www.steadyhealth.com forums were found to match the Adverse Drug Reactions published in Drug Package Inserts for several drugs. In addition, some novel adverse side-effects, which can act as early indicators of Adverse Drug Reactions, were also identified.

## Methods

**Figure 1:**
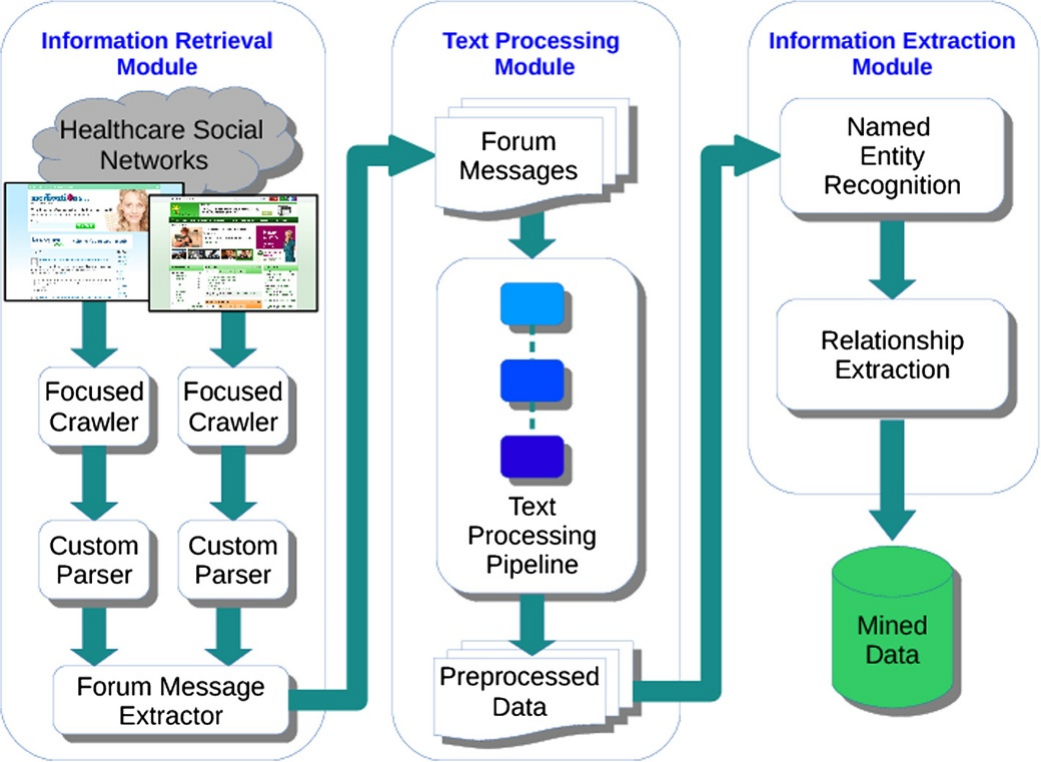
**System architecture of an adverse drug reaction mining system.**

Text Mining systems are primarily used in the discovery and extraction of knowledge from unstructured text data [40]. Figure 1 presents the architecture of our Text Mining system used for extracting Drug-Side Effects relationships from online healthcare forums. It primarily consists of the following 3 modules:

- Information Retrieval Module to create a collection of relevant documents
- Text Processing Module to preprocess text in the collected documents to facilitate extraction
- Information Extraction Module to extract information of interest from preprocessed texts

### Information retrieval module

The Information Retrieval module consists of a system that is responsible for extracting relevant documents or data sources from which we are interested to extract useful information. Some of the common approaches for data collection include: collecting results from search engines, using web services to access content or creating web crawlers to extract relevant pieces of information from web pages. Using focused web crawlers [41] is more suitable for accessing content from online healthcare forums in comparison to the conventional snowball crawlers as they help in limiting the data collected to be within the targeted websites. The crawlers are usually built specific to a website as they need to parse through the presentational and navigational elements of each website to extract the relevant content. A crawler built for crawling a healthcare forum would identify all the distinct threads in a forum, parse through the different pages of each thread to extract all messages posted on that thread. For this study, we built focused web crawlers to extract data from two different sources: http://www.medications.com and http://www.steadyhealth.com. The web crawler was written in Java using JSoup [42], a Java HTML parser library. In all, about 8065 posts where collected across 1439 threads from http://www.medications.com based on content available in June 2012. Similarly, about 29981 online posts were collected from about 11878 threads spanning across 29 independent forums from http://www.steadyhealth.com in October 2012. The dataset from the medications.com was primarily used in the training and validation of the HMM classifier, while the one from steadyhealth.com was used in the analysis of the mined side-effects.

It is to be noted that this study did not involve any experimental research on humans or animals, hence an approval from an ethics committee was not applicable in this regard. The data collected from the online healthcare forums are publicly available data and no personally identifiable information of the forum users were collected or used for this study.

### Text processing module

**Figure 2:**
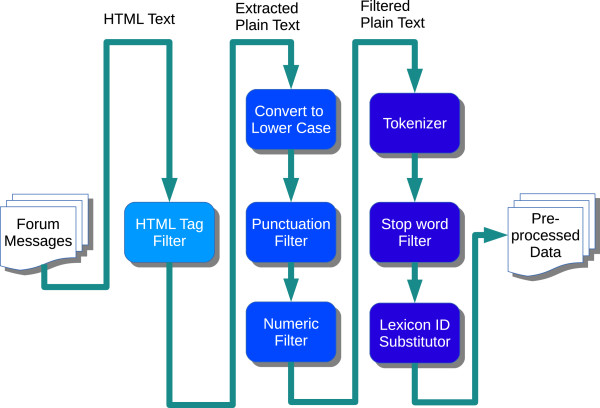
**Text processing module.**

The Text Processing module is used to extract textual units from document collections and process them into a format suitable for use by the Information Extraction module. Typically, this module is comprised of several Natural Language Processing (NLP) tools linked together as a pipeline for processing text data. Figure 2 presents the text processing steps in our system. In order to have a robust system that is not affected by the semantics of the language, we do not include techniques like part-of-speech tagging, stemming or word sense disambiguation. First, the crawled web document collection is parsed to extract unique thread names and associated messages. Each of these messages are then processed to remove HTML tags, converted to lower case and run through filters to remove unwanted punctuation and raw numerical data. The resulting text is then tokenized, filtered of common stop words and substituted with respective lexicon identifiers for ease of processing in the information extraction stage.

### Information extraction module

**Figure 3:**
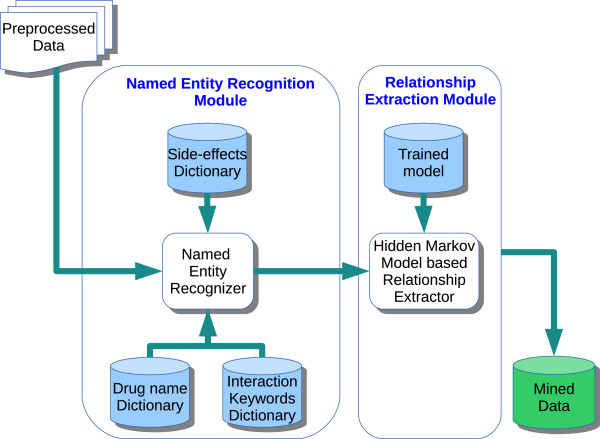
**Information extraction module.**

The Information Extraction [43] module is used to identify entities of interest in the preprocessed data and extract possible relationships between them. As shown in Figure 3, it consists of the *Named Entity Recognition* (NER) and *Relationship Extraction* (RE) sub-modules.

Named Entity Recognition helps to identify entities of interest in a given text. In our scenario, the entities of interest would be names of drugs, terms denoting side-effects and keywords or phrases that indicate a relationship between the drug and a side-effect. Lack of standard naming conventions usually make this step a challenging task. In general, NER can be performed using rule-based or lexicon-based methods. [44] and [45] present some recent approaches used as a part of the challenges like 2009 i2b2 Clinical NLP Challenge and 2013 BioCreative CHEMDNER for extracting drug names in the clinical domain.

When using the lexicon-based methods for performing NER, the choice of vocabulary that is used to create the dictionary entries has a significant impact on the performance of the NER module. So it becomes necessary that the vocabulary of the dictionary reflect the vocabulary of the target corpus to be mined. In this regard with the messages in the online forums being the target corpus in our approach, we would need to construct a dictionary that would reflect the vocabulary observed in such forum messages. Majority of the users of such online forums, do not possess the medical background to use technical terms to identify the drug names and side-effects. The comprehensive drug dictionaries, such as those used as a part of the i2b2 Clinical NLP and BioCreative challenges, tend to use technical terms to identify the mentions of drug names, which do not form the vocabulary of the average forum user. So including comprehensive drug name dictionaries may not necessarily improve the performance of the NER module. It is in this regard we construct a custom drug dictionary with only minimal entries that would reflect the vocabulary of an average user.

The dictionary of drug names was created by crawling the drug lists available as a part of drugs.com [46] website, which was filtered to create a list of about 760 common drug names. SIDER, the side-effects resource, was used as the primary resource for creation of the dictionary of side-effects. In all about 1390 side-effect terms were created from this resource. In order to identify keywords and phrases that denote the cause of a side-effect by a drug, a frequency analysis of the *n*-grams was performed on the text corpus. High frequency *n*-grams, with counts more than 20, where identified for *n* varying between 2 and 5. The filtered list was manually reviewed to extract a final list of about 45 keywords and phrases that denote the causal relationship of a drug causing a side-effect. Table 1 presents the list of the extracted keywords and phrases.

**Table 1:** **List of keywords and phrases denoting a side-effect due to a drug**

After having	Found out	Reaction to
After stopping	Found that	Result of
Because of this	Had a problem	Side affects
Caused by	Have been getting	Side effect
Cause of	Have been having	Side effects
Developed	Have noticed	Since i got
Due to	Have started	Since i stopped
Effects from	I am having	Since then
Effects of	I am starting	Started getting
Ever since	I now have	Started having
Experienced	Made me feel	Started noticing
Experiencing	Makes me feel	Started taking
Feeling	Now i have	Started to
Feel like	Problems with	Starting to feel
Felt like	Problem with	Was causing

The Relationship Extraction module is used to identify presence of relationships between the named entities in a given text. In general, several techniques including rule-based, statistical co-occurrence, and natural language processing methods have been employed for this purpose. We make use of Hidden Markov Model (HMM), a supervised machine learning approach, to predict the presence of relationship between a drug and an adverse side-effect.

If a message contains only a drug name and side-effect mention, it is not sufficient to denote a positive ADR. There needs to be some form of causal relationship that clearly associates the drug with the side-effect. It is in this regard that the keywords identified by the HMM are used to capture the causal relationship. As a part of the training, the HMM is trained on positive samples where it learns the association between the drugs and side-effects through the presence of keywords and uses this information for relationship prediction on the test data set.

### Hidden Markov model

A Hidden Markov model is a statistical model in which the system being modeled is assumed to be a Markov process with hidden states. The outputs of the hidden states are observable and are represented as probabilistic functions of the state. In general, a HMM is defined using the following parameters:

- N: Number of states in the HMM
- M: Number of observation symbols in the HMM
- A = [a _*ij*_]: N by N state transition probability matrix
- B = b _*j*_(m): N by M observation probability matrix
- *Π* = [ *π*_*i*_]: N by 1 initial state probability vector

HMMs have primarily been used to model sequence data like speech utterances in speech recognition [47] and Part-of-Speech tagging [48]. They have also been successfully used for Information extraction [49] and Named Entity Recognition [50]. The success of HMMs in these tasks has motivated us to explore the possibility of using them to perform Relationship Extraction. Jahmm [51], the Java Hidden Markov Model library, was used for implementing the Hidden Markov Model.

### Data sources

#### Medications.com

**Figure 4:**
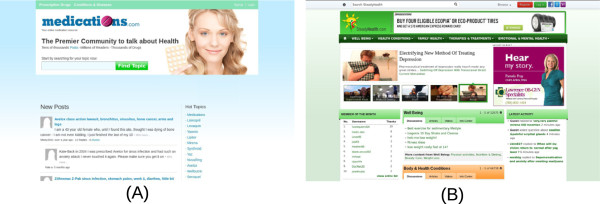
**Healthcare discussion forums. (A)** http://www.medications.com is an online forum for discussing health focusing on drugs and conditions **(B)** http://www.steadyhealth.com is an online community for discussing health.

Medications.com [24] (Figure 4A) is an online forum for discussing drugs, conditions, procedures and other information related to the general well being. It contains tens of thousands of user generated posts relating to thousands of drugs. It contains topics that are organized based either on the name of the drugs or the condition that is being treated. The posts in this forum provide an ideal source for extracting drugs and their side-effects. The data from this source was used as a part of the pilot study [52].

#### SteadyHealth.com

SteadyHealth.com [25] (Figure 4B) is an online health-related community intended for users to educate themselves on health related aspects, share their experiences and exchange access to health-related resources. In this forum, users are able to post questions, comments, and respond to messages from others regarding medical and health related issues. Their discussion boards spread messages and topics over 30 different categories with more than 150 forums providing access to both registered members and guests. It is a rapidly growing health community with more than 65,000 registered members. This forum can provide an ideal source for collecting health related information across several categories including general well being, body and health conditions, family health, therapies and treatments, and emotional and mental health.

### Forum messages

**Figure 5:**
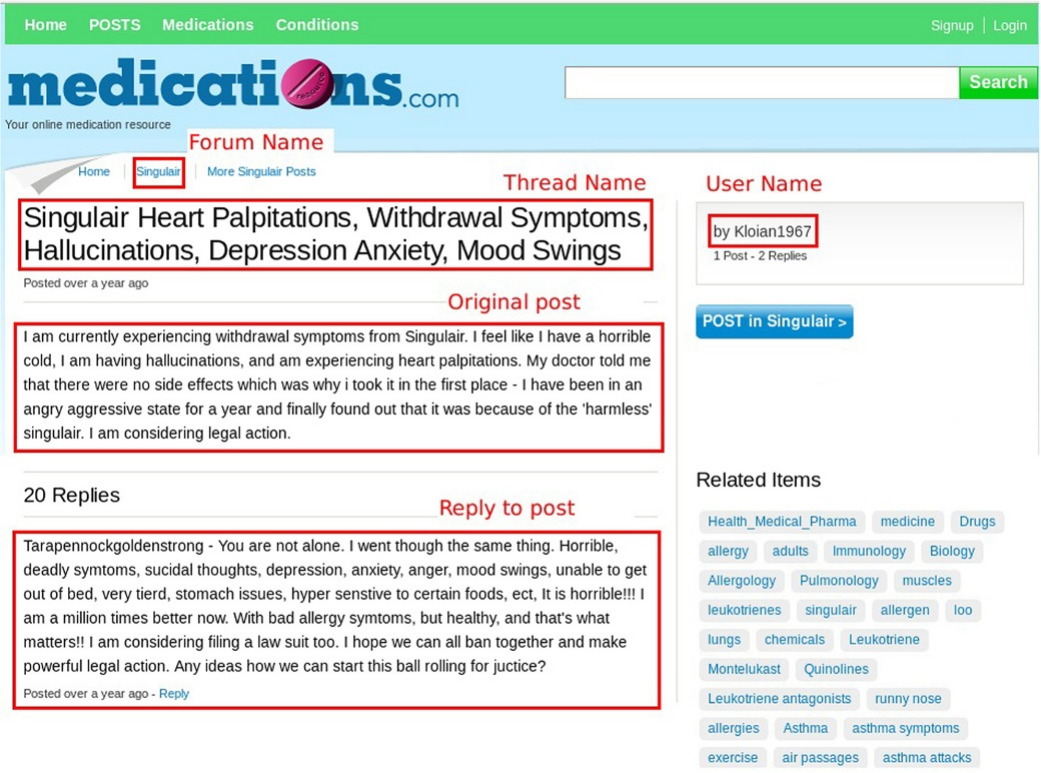
**Sample messages posted on** http://www.medications.com.

**Figure 6:**
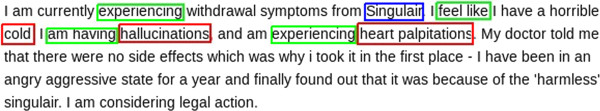
**Message highlighting the mention of adverse drug reactions.** The message highlights the mention of Adverse Drug Reactions as a part of the Online Healthcare Forums. The drug name mentions are highlighted by a blue border, the keywords connecting the drug to an adverse effect are highlighted by a green border and the side-effects are highlighted by a red border.

**Figure 7:**
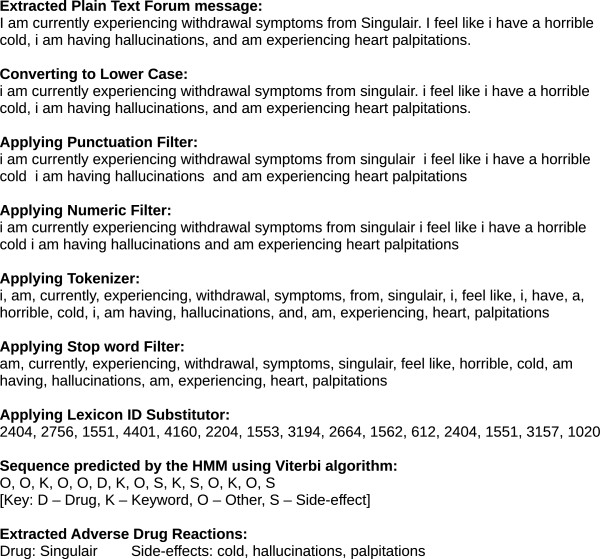
**A sample message being processed through the text processing and information extraction module.**

A typical forum may consist of topics or discussion boards which help categorize the nature of discussions. For e.g., in Medications.com each drug has a topic or discussion board of its own. Under each of these discussion boards there would be multiple threads that are created to talk about specific issues about the drug. Each of these threads in turn may have multiple posts or messages where other users may comment or provide feedback on the issues raised by the original poster. As an example, Figure 5 provides a screen shot of a thread under the discussion board for the drug Singulair. It presents a message posted by the user, identified by the name Kloian1967, regarding the side-effects of Singulair. It also contains a follow up reply made by another user in response to the original poster. Though the screenshot provides a simple interface containing the plain text information, behind the scenes, the HTML used to present this content may be mixed with Javascript and other presentational elements including CSS. Figure 6 presents the annotated version of the message with highlights identifying the mentions of all the different entities that denote the presence of an adverse drug side-effect. Figure 7 presents the transformations of the sample message as it passes through the different stages in the text preprocessing pipeline before being passed to the Information Extraction module to extract ADRs.

### Message model

**Figure 8:**
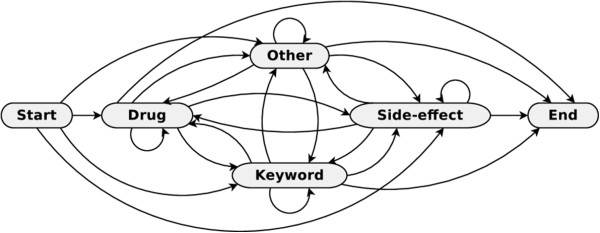
**Model of a forum message containing drug and side-effect information.** A forum message containing mentions of drug and its associated side-effects is modeled using an Hidden Markov Model. The presented model represents an ergodic HMM where every state is connected to every other state including itself and allows for transitions between each of them. This models a forum post where the mentions of drug names, side-effects, keywords and other words can appear in any sequence with possibilities for repetition.

A typical message from a healthcare forum discussing adverse drug reactions would consist of multiple sentences that describe the drug and its side-effects along with some keywords that establish the cause-and-effect relation between them. Most often this information is presented in a sequential order, starting with a drug that was prescribed and the reactions that occurred once the patient started taking the drug. Since the information can span across several sentences the model is used to represent the message as a whole instead of a single sentence. To facilitate the extraction of this information, we create a model that represents a message with the drug name, relation keyword and side-effect as its hidden states. In addition, the message may also contain words that don’t necessarily convey any useful information which are represented by the ‘other’ state. The actual sequence of words appearing in the sentences are the observations emitted from these four states. In order to make the model more robust, we allow the three named entities to occur in any order within the message. Figure 8 presents the states and transitions of a HMM that is used to model a typical online healthcare forum message describing a drug and its side-effects. The Start and End states merely denote that the sequence of words can both start and end with either of the four states and are not part of the hidden states of the HMM.

### Data sets

The messages crawled from http://www.medications.com were used for creating the train and test data sets used in the evaluation of the HMM classifier. Due to the large number of messages a two phase approach was carried out for annotating the messages. First an automated annotation was carried out on the entire corpus of the 8065 messages, by making use of the dictionaries for drug names, side-effects and keywords. Messages with only one drug name mention and with all three entity types identified were flagged to belong to the positive data set, while the rest formed the negative data set. In all, the automatically annotated data set consisted of 2091 positive and 5974 negative samples giving about 25% chance of finding a positive ADR in the dataset. Of the 2091 automatically annotated positive message samples, 500 messages were manually reviewed and annotated to form the positive training set. Similarly another 1500 negative samples were picked to form the negative training set thereby maintaining a ratio of about 25%-75% between the positive and negative samples.

### Training

**Figure 9:**
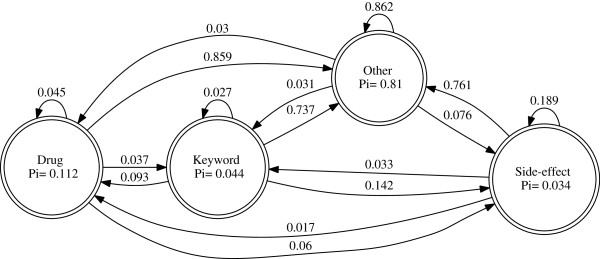
**A trained HMM displaying the initial state and state transition probabilities learnt from the training set.**

The manually annotated training set of 2000 messages is used to train the HMM classifier. Since the Baum-Welch algorithm that is used for training the HMM is only capable of finding the locally optimal solution, it is important that the HMM be initialized with probabilities that are closer to probabilities of the learnt model. We do this by using the manually annotated training set where we have both the observation and its corresponding state annotation. By counting the frequencies of the number of times the observations start in a particular state, number of times transitions occur between states and number of times an observation is emitted from a state we are able to compute the probabilities for the initial starting state of the HMM, the transition probabilities and the emission probabilities. With these values forming the initial model of the HMM we go on to train this model with the sequences from the training data set using the Baum-Welch algorithm. Figure 9 shows the trained hmm with initial state and state transition probabilities.

### HMM based classifier

Based on the probabilities learnt from the training set, the Viterbi decoding algorithm is then used to predict the hidden states for the observed sequence data in the testing set. Based on the identified states, if a message contains all the three states, it is flagged to have a positive drug/side-effect relationship. Even though there can be multiple occurrences of the same drug name or a side-effect in a forum message, only one such instance of each entity is used as a part of the mined information. Since the prediction from the HMM classifies the messages as either having a drug/side-effect relation or not, the results can be presented using the typical True Positive (TP), False Positive (FP), True Negative (TN) and False Negative (FN) measures. Based on these measures, the performance of the HMM prediction model is computed using the following standard metrics of Precision, Recall, F-Score and Accuracy.1234

### Baseline classifier

In order to compare the performance of the HMM based classifier, a Baseline classifier based on the co-occurrence frequencies of drug names and side-effects was built. Co-occurrence statistics are a very common measure for identifying associations and relationships between words [53]. For all the 760 drug names and 1390 side-effects in the dictionary, a co-occurrence frequency map was constructed based on their occurrence in a forum message. Within a forum message, even though a drug name and a side-effect occur more than one time, their co-occurrence count was still considered to be one, in order to prevent a single forum post from influencing the co-occurrence frequencies. Similar to the HMM based classifier, the Baseline Classifier is also given the same training data set, from which the drug/side-effect co-occurrence statistics are computed. Given a test set, the Baseline classifier extracts every unique drug/side-effect pair in the given message and flags them to have a positive relationship in case their co-occurrence frequency was computed to be greater than zero. The same metrics used in the evaluation of the HMM-based classifier are also used in case of the Baseline classifier.

## Results and discussion

In order to compare the performance of the classifiers we do a 10-fold cross-validation on the 2000 sample manually annotated training dataset. Table 2 presents the results of a single run of the 10-fold cross-validation for both the Baseline and HMM classifiers. In general, the HMM-based classifier performed better with an average F-Score of 0.76 in comparison to the Baseline classifier which yielded an average F-Score of 0.575. It is evident that the Baseline classifier performs poorly in comparison to the HMM classifier as both its the False Positive and False Negative values are higher. The higher False Negatives can be attributed to the fact that the baseline classifier is not able to predict ADR relationship for drug/side-effect combinations that it has not seen before. The HMM-based classifier, in contrast, is able to predict such relationships, even in cases where positive ADRs between a specific drug and its side-effect were not available as a part of the training set. It is in this regard that the HMM classifier is capable of extracting some novel drug/side-effect information as well.

**Table 2:** **Results of a 10-fold cross-validation run for baseline and HMM classifiers**

					Baseline classifier					
**Iteration**	**Train set**	**Test set**	**True**	**False**	**True**	**False**	**Precision**	**Recall**	**F-Score**	**Accuracy**
			**positive**	**positive**	**negative**	**negative**				
1	1761	196	27	8	135	26	0.771	0.509	0.614	0.827
2	1761	196	25	15	137	19	0.625	0.568	0.595	0.827
3	1761	196	35	14	125	22	0.714	0.614	0.660	0.816
4	1761	196	27	14	130	25	0.659	0.519	0.581	0.801
5	1761	196	28	9	130	29	0.757	0.491	0.596	0.806
6	1761	196	27	21	131	17	0.563	0.614	0.587	0.806
7	1761	196	23	17	137	19	0.575	0.548	0.561	0.816
8	1761	196	27	17	128	24	0.614	0.529	0.568	0.791
9	1761	196	18	12	140	26	0.6	0.409	0.486	0.806
10	1763	194	28	10	128	28	0.737	0.5	0.596	0.804
					**HMM classifier**					
**Iteration**	**Train set**	**Test set**	**True**	**False**	**True**	**False**	**Precision**	**Recall**	**F-Score**	**Accuracy**
			**positive**	**positive**	**negative**	**negative**				
1	1761	196	42	8	136	10	0.84	0.808	0.824	0.908
2	1761	196	30	10	142	14	0.75	0.682	0.714	0.878
3	1761	196	50	10	123	13	0.833	0.794	0.813	0.883
4	1761	196	29	5	142	20	0.853	0.592	0.699	0.872
5	1761	196	37	14	135	10	0.725	0.787	0.755	0.878
6	1761	196	40	9	140	7	0.816	0.851	0.833	0.918
7	1761	196	37	11	131	17	0.771	0.685	0.725	0.857
8	1761	196	44	8	131	13	0.846	0.772	0.807	0.893
9	1761	196	29	16	143	8	0.644	0.784	0.707	0.878
10	1763	194	34	9	135	16	0.791	0.68	0.731	0.871

In general, co-occurrence of a drug name and a side-effect does not necessarily imply presence of a positive ADR. It is for this reason the False Positives for the Baseline classifier are higher. There needs to be a clear indication of a causal relationship that shows a drug is responsible for a side-effect. It is in this regard that the additional keyword information used by the HMM classifier is capable of identifying the causal relationship between the drug and the side-effect. The False Positives in case of the HMM classifier were identified to be caused primarily due to the lack of distinction between the symptoms that a drug is treating and the side-effects it causes. We believe we could address this by maintaining a list of symptoms for which a drug is prescribed and eliminate them from the list of side-effects identified to improve the accuracy of the classifier.

The accuracy of a classifier also depends upon the components available as a part of the Text Processing module and the sizes of the dictionaries used as a part of the Information Extraction module. We conducted some additional experiments in order analyze the effect of these on the classification accuracy.

**Figure 10:**
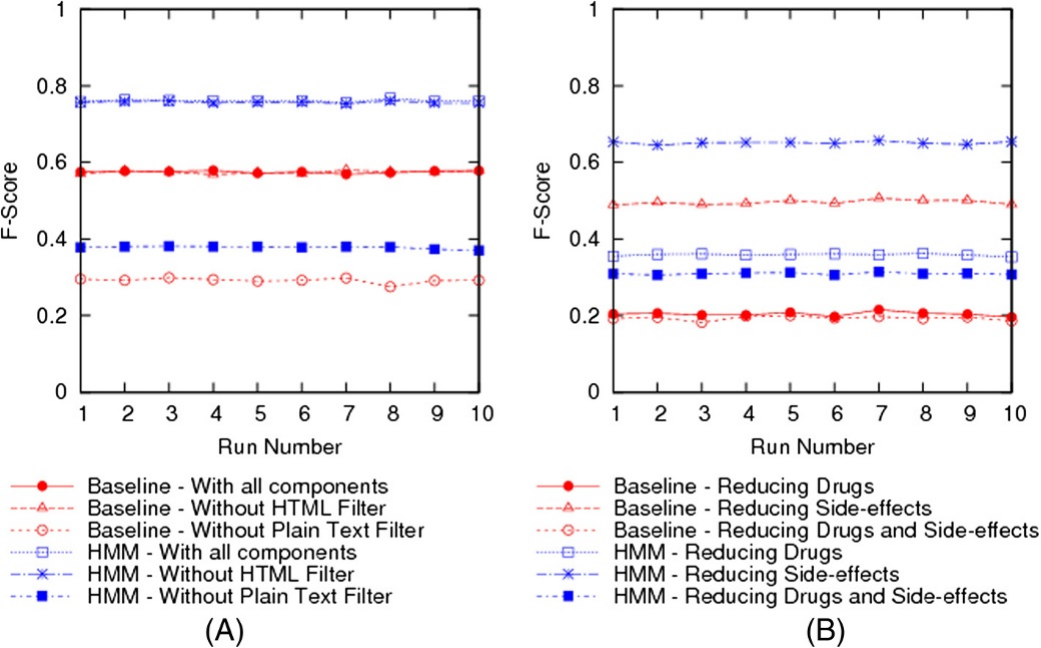
**Graphs comparing the F-Score measure of the baseline classifier against the HMM classifier by (A) varying the components used and (B) varying the dictionary sizes.**

As a part of the experiments analyzing the effect of text processing components, we tried removing the components that transitioned the text through the different stages of the Text Processing pipeline as shown in Figure 2. We first removed the HTML Tag filter component, which allowed text containing HTML to flow into the next stage of the text processing instead of the extracted plain text and performed the classification. We then included the HTML filter back, but then removed the second stage of the Text processing pipeline containing conversion of text to lower case, punctuation filter and numeric filter, and again performed the classification. We ran these experiments for both the Baseline and HMM Classifiers and then compared them against the system that used all the components. Tables 3 and 4 present the results of these experiments which include the mean values of Precision, Recall and F-Score computed across 10 different runs of the 10-fold cross-validation for the Baseline and HMM classifiers, respectively. Figure 10(A) presents a plot of the F-Score values of the Baseline and HMM Classifiers across 10 different runs with the variations in the components used.

**Table 3:** **Results of baseline classification with varying components across 10 runs of 10-fold cross-validation**

		With all components			No HTML filter			No plain text filter	
Run	Mean	Mean	Mean	Mean	Mean	Mean	Mean	Mean	Mean
	Precision	Recall	F-Score	Precision	Recall	F-Score	Precision	Recall	F-Score
1	0.648	0.519	0.575	0.643	0.514	0.570	0.503	0.211	0.295
2	0.648	0.527	0.577	0.645	0.530	0.578	0.513	0.206	0.292
3	0.652	0.519	0.576	0.649	0.518	0.575	0.510	0.213	0.299
4	0.654	0.523	0.579	0.645	0.510	0.568	0.494	0.212	0.294
5	0.639	0.522	0.572	0.648	0.522	0.573	0.502	0.207	0.290
6	0.640	0.525	0.575	0.646	0.515	0.570	0.511	0.207	0.292
7	0.643	0.513	0.569	0.653	0.524	0.580	0.504	0.213	0.298
8	0.646	0.519	0.573	0.652	0.516	0.575	0.475	0.195	0.275
9	0.651	0.523	0.577	0.651	0.517	0.575	0.515	0.206	0.291
10	0.652	0.522	0.578	0.656	0.520	0.578	0.513	0.206	0.292

**Table 4:** **Results of HMM classification with varying components across 10 runs of 10-fold cross-validation**

		With all components			No HTML filter			No plain text filter	
Run	Mean	Mean	Mean	Mean	Mean	Mean	Mean	Mean	Mean
	Precision	Recall	F-Score	Precision	Recall	F-Score	Precision	Recall	F-Score
1	0.779	0.742	0.758	0.777	0.742	0.758	0.636	0.274	0.378
2	0.785	0.743	0.763	0.776	0.746	0.759	0.634	0.273	0.380
3	0.782	0.746	0.762	0.785	0.739	0.760	0.646	0.274	0.381
4	0.778	0.747	0.761	0.777	0.736	0.755	0.640	0.272	0.380
5	0.779	0.746	0.761	0.780	0.738	0.757	0.640	0.271	0.380
6	0.787	0.743	0.761	0.781	0.741	0.758	0.626	0.274	0.378
7	0.773	0.741	0.756	0.778	0.733	0.753	0.634	0.274	0.380
8	0.784	0.755	0.767	0.788	0.744	0.761	0.635	0.271	0.379
9	0.783	0.743	0.760	0.779	0.735	0.755	0.621	0.270	0.373
10	0.790	0.742	0.760	0.785	0.732	0.755	0.623	0.265	0.370

From the tables it can be seen that removing the HTML filter component had almost no impact on the F-Score for both the Baseline and HMM Classifiers. This can be attributed to the fact that most of the forum messages did not contain a lot of embedded HTML tags in their content and hence did not add as much ‘noise’ to reduce the classification accuracy. However, removing the plain text filtering component did have a big impact on the classification accuracy. Removing this component reduced the F-Score of the Baseline classifier from 0.575 to 0.292 on average. Similarly, for the HMM Classifier the F-Score was reduced from 0.76 to 0.378 on average. The main task of the plain text filtering component is to normalize text by converting to lower case and filtering punctuation and numeric values. Without this normalization, there would be several variations of a word (same word with different cases Eg: ‘Lipitor’, ‘lipitor’; and same word ending with different punctuation, e.g. ‘headache,’ and ‘headache’.). Such variations in turn affect the performance of the Named Entity Recognition module which in turn reduces the prediction accuracy of the classifier.

The third stage of the Text Processing pipeline contains the Tokenizer, Stop word filter and Lexicon ID substitutor. The Tokenzier and Lexicon ID Substitutor are components which are necessary to transform the data into a format that can be used by the classifiers for training and prediction, without them it would not be possible to obtain classification results. Hence, we tried removing only the Stop word filter to evaluate the impact on classification results. From the experiments we found that removing the Stop word filter almost had no impact on the classification accuracy, as on average, the Baseline classifier yielded an F-Score of 0.573 and the HMM Classifier yielded an F-Score of 0.759, which are very close to the average prediction accuracy of the classifiers with all components. The Baseline classifier is not affected by the stop word filter as it is primarily dependent only on the co-occurrence of drugs and side-effects, while in case of the HMM Classifier the unfiltered stop words would simply be flagged as words belonging to the ‘Other’ state. Having the stop word filter however does help in reducing the amount of processing needed as only fewer words need to be processed by the classifiers.

In order to evaluate the effect of dictionary sizes on the classification results, we tried reducing the size of the Drug names and Side-effect dictionaries to half their original size and then performed the classifications. Tables 5 and 6 present the mean values of Precision, Recall and F-Score for the runs of 10-fold cross validation by reducing the size of the Drug names dictionary only, Side-effects dictionary only and both Drug names and Side-effects dictionaries. Figure 10(B) presents a plot of the F-Score values of the Baseline and HMM Classifiers across 10 different runs with the dictionary sizes reduced to half their original size. From the tables, it can be seen that reducing the size of the Drug names dictionary had the major impact where the F-Score was reduced to 0.204 on average for the Baseline Classifier and 0.359 on average for the HMM Classifier. Reducing the size of the Side-effects dictionary had a marginal impact with the F-Score being reduced to 0.496 on average for the Baseline Classifier and 0.651 on average for the HMM Classifier. As expected, reducing the sizes of both dictionaries had the most impact with the F-Score being reduced to 0.193 on average for the Baseline Classifier and to 0.309 on average for the HMM Classifier. By reducing the size of the dictionaries, we limit the number of drugs or side-effects identified by the Named Entity Recognition module which in effect reduces the prediction accuracy of the classifier by introducing more False Negatives. In general, we would want to keep the size of the dictionaries to match the vocabulary of the average forum user in order to achieve better coverage and good prediction accuracy.

**Table 5:** **Results of varying the dictionary sizes for baseline classification**

	Drug names dictionary size			Side-effects dictionary size			Both the dictionary sizes		
	reduced to half			reduced to half			reduced to half		
Run	Mean	Mean	Mean	Mean	Mean	Mean	Mean	Mean	Mean
	Precision	Recall	F-Score	Precision	Recall	F-Score	Precision	Recall	F-Score
1	0.535	0.128	0.204	0.645	0.394	0.489	0.575	0.117	0.192
2	0.543	0.128	0.206	0.649	0.405	0.495	0.566	0.119	0.195
3	0.561	0.125	0.201	0.643	0.401	0.490	0.574	0.110	0.182
4	0.538	0.126	0.201	0.664	0.398	0.492	0.574	0.120	0.198
5	0.527	0.131	0.208	0.654	0.412	0.501	0.569	0.123	0.200
6	0.506	0.124	0.197	0.644	0.401	0.493	0.577	0.117	0.193
7	0.541	0.137	0.215	0.665	0.412	0.506	0.566	0.120	0.197
8	0.527	0.130	0.206	0.660	0.407	0.500	0.592	0.117	0.192
9	0.524	0.131	0.203	0.657	0.408	0.501	0.599	0.119	0.196
10	0.511	0.125	0.196	0.644	0.397	0.490	0.551	0.114	0.186

**Table 6:** **Results of varying the dictionary sizes for HMM classification**

	Drug names dictionary size			Side-effects dictionary size			Both the dictionary sizes		
	reduced to half			reduced to half			reduced to half		
Run	Mean	Mean	Mean	Mean	Mean	Mean	Mean	Mean	Mean
	**Precision**	**Recall**	**F-Score**	**Precision**	**Recall**	**F-Score**	**Precision**	**Recall**	**F-Score**
1	0.684	0.242	0.355	0.793	0.556	0.653	0.718	0.201	0.309
2	0.692	0.246	0.360	0.781	0.554	0.645	0.730	0.196	0.306
3	0.691	0.249	0.361	0.785	0.559	0.651	0.761	0.197	0.309
4	0.670	0.245	0.358	0.787	0.559	0.652	0.734	0.200	0.311
5	0.683	0.248	0.360	0.788	0.560	0.652	0.747	0.200	0.312
6	0.685	0.247	0.361	0.790	0.557	0.650	0.724	0.200	0.306
7	0.682	0.248	0.359	0.793	0.564	0.657	0.741	0.202	0.314
8	0.687	0.247	0.362	0.784	0.556	0.650	0.742	0.200	0.309
9	0.684	0.245	0.358	0.786	0.553	0.647	0.728	0.200	0.310
10	0.685	0.244	0.353	0.787	0.564	0.654	0.710	0.200	0.307

### Mining adverse side-effects

In all, side-effects for about 168 unique drugs were mined from the medications.com data set. There were about 30 drugs for which the HMM based Text Miner was able to extract side-effect information from more that 10 forum messages. Similarly, there were about 316 unique drugs for which side-effects were mined from the steadyhealth.com data set with about 50 drugs having information mined from more than 10 forum messages. Table 7 presents statistics on the mined side-effect information for some of the drugs having the most number of messages.

**Table 7:** **Mined side-effect counts for some high frequency drugs**

Medications.com				
**Drug**	**Number**	**Total**	**Number**	**Number of**
**name**	**of forum**	**Number of**	**of unique**	**side-effects**
	**messages**	**side-effects**	**side-effects**	**occurring**
	**mined**	**mined**	**mined**	**> 5 times**
Lisinopril	255	939	240	48
Prednisone	111	539	180	24
Singulair	103	446	142	19
Kenalog	84	263	110	11
Topamax	60	266	98	10
**Steadyhealth.com**				
**Drug**	**Number**	**Total**	**Number of**	**Number of**
**name**	**of forum**	**Number of**	**unique**	**side-effects**
	**messages**	**side-effects**	**side-effects**	**occurring**
	**mined**	**mined**	**mined**	**> 5 times**
Adderall	180	530	173	27
Cortisone	170	507	159	20
Effexor	141	478	134	19
Suboxone	108	296	91	9
Zoloft	103	406	123	16

### Comparison to adverse drug reactions in drug package inserts

The side-effects extracted from both medications.com and steadyhealth.com data sets were then compared with the actual side-effects as reported in the Drug Package Inserts. Table 8 presents this comparison for the top four drugs with the highest number of posts from both the forums. In addition to the name of the drug and what it is prescribed for, the table also lists the set of most common Adverse Drug Reactions that have been reported in the Drug Package Inserts, the set of reported Adverse Drug Reactions that have been mined from the forum messages and a set of novel side-effects mined from the forum messages. The percentage in brackets for the mined adverse side-effects denotes the percentage of occurrence of that side-effect among all the identified side-effects for a drug as mined from the forum messages. From the mined data it can be seen that HMM based Text Miner was able to extract adverse drug reactions that are in agreement with the adverse drug reactions as reported in the Drug Package Inserts.

**Table 8:** **Comparison of mined drug adverse reactions with those reported in drug package inserts**

Drug Name	Prescribed for	Common ADRs in	Common ADRs mined	Novel side-effects mined
		Drug Package Inserts	from medications.com	from medications.com
Lisinopril	High blood pressure	Headache, dizziness, cough,	Cough (12.57%), dizziness (2.77%),	Hearing loss (0.53%), hair
	(hypertension),	fatigue, rash, diarrhea, nausea,	headache (1.81%), fatigue (1.49%),	loss (0.53%), shingles (0.43%),
	congestive heart failure,	cramps	cramps (1.38%), diarrhea (0.96%),	fits (0.32%)
	improve survival after a		nausea (0.75%), rash (0.43%)	
	heart attack			
Prednisone	Allergic disorders,	Anxiety, dizziness, depression,	Anxiety (5.57%), insomnia (3.15%),	Hives (1.3%), acid reflux
	skin conditions,	insomnia, headache, nausea,	depression (2.97%), dizziness (2.41%),	(0.37%), avascular necrosis
	ulcerative colitis,	moon face, elevation in blood	mood swings (2.41%), weight gain	(0.37%), dry mouth (0.37%),
	arthritis, lupus,	pressure, behavioral and	(1.86%), nausea (1.3%), moon face	
	breathing disorders	mood changes, weight gain	(1.11%)	
Singulair	Asthma, allergic rhinitis	Upper respiratory infection,	Headache (2.02%), infection (1.12%),	Seizure (6.28%), depression
		fever, headache, pharyngitis,	cough (1.12%), fever (0.90%),	(3.59%), nightmares (3.36%),
		cough, abdominal pain,	diarrhea (0.45%), sinusitis (0.45%),	aggression (2.91%), mood
		diarrhea, influenza,	inflammation (0.45%)	swings (2.02%), suicide
		rhinorrhea, sinusitis		(1.35%), suicidal thoughts
				(0.9%)
Topamax	Seizures, migraine	Anorexia, paresthesia (tingling),	Tingling (5.64%), weight loss (4.14%),	Hair loss (3.01%), depression
	headaches	fatigue, nervousness, weight	memory loss (3.76%), numbness	(2.26%), stress (1.88%),
		decrease, somnolence,	(2.26%), dizziness (2.26%), tired	aches (1.88%), anxiety (1.13%),
		dizziness, infection, flushing,	(1.88%), sleepy (1.13%)	diarrhea (1.13%), dry mouth
		psychomotor slowing,		(1.13%), itching (1.13%)
		difficulty with memory		
**Drug Name**	**Prescribed for**	**Common ADRs in**	**Common ADRs mined**	**Novel side-effects mined**
		**Drug Package Inserts**	**from steadyhealth.com**	**from steadyhealth.com**
Adderall	Narcolepsy and	Palpitations, elevation of blood	Depression (6.04%), weight loss	Anxiety (4.53%), fatigue
	attention deficit	pressure, sudden death,	(5.10%), headache (3.97%), dizziness	(3.39%), addiction (2.45%),
	hyperactivity	myocardial infarction, dryness	(2.64%), dry mouth (1.70%), insomnia	mood swings (1.89%),
	disorder (ADHD)	of the mouth, diarrhea, weight	(1.51%), constipation (1.13%), loss	vomiting (1.32%), nausea
		loss, constipation, rash,	of appetite (1.13%), death (0.94%),	(1.13%), hallucinations
		restlessness, dizziness,	seizures (0.75%), high blood	(0.75%)
		insomnia, depression,	pressure (0.57%), restlessness (0.57%)	
		headache, seizures		
Cortisone	Allergic disorders,	Allergic reactions, cardiac	Headache (2.37%), allergies (1.97%),	Anxiety (2.96%), cramps
	skin conditions,	arrest, hypertension, acne,	nausea (1.97%), weight gain (1.97%),	(2.57%), bleeding (2.37%),
	ulcerative colitis,	cutaneous and subcutaneous	depression (1.58%), insomnia (1.38%),	bleeding (2.37%), back pain
	arthritis, lupus,	atrophy rash, increased	high blood pressure (1.38%), acne	(2.17%), dizziness (1.97%),
	psoriasis, or	appetite, depression, mood	(1.18%), atrophy (0.99%), rash (0.79%)	numbness (1.18%), diarrhea
	breathing disorders	swings, nausea, headache,		(0.99%)
		insomnia, weight gain		
Effexor	Major depressive	Insomnia, dizziness, dry mouth,	Dizziness (6.07%), headache (3.35%),	Weight gain (3.97%), acne
	disorder, anxiety,	nausea, headache, sweating,	nausea (2.09%), sweating (1.26%),	(1.26%), shocks (1.26%),
	and panic disorder	chills, vomiting, diarrhea,	insomnia (1.05%), vomiting (1.05%),	hives (1.05%), mood swings
		tachycardia	chills (1.05%), diarrhea (1.05%),	(0.84%)
			tachycardia (0.84%)	
Suboxone	Narcotic (opiate)	Headache, vomiting, nausea,	Pain (17.23%), insomnia (2.7%),	Anxiety (5.07%), tired (4.39%),
	addiction	hyperhidrosis (sweating),	depression (2.7%), chronic pain (2.03%),	restlessness (4.05%), chills
		insomnia, constipation, pain,	sweats (2.02%), headaches (1.01%)	(3.72%), weight gain (1.69%),
		depression and peripheral		runny nose (1.35%)
		edema		

### Case studies from the mined data

Prednisone was one of the drugs with the most number of messages containing adverse side-effect mentions, about 111, in the Medications data set. Using the HMM Text Miner we were able to extract about 180 different side-effect mentions from these messages. From the listing in Table 8 it can be seen that most of the reported adverse reactions like anxiety, dizziness, insomnia, depression, weight gain and moon face have been identified. In addition we were able to extract some novel side-effects like hives, acid reflux, avascular necrosis and dry mouth in relation to use of Prednisone. One of the identified novel side-effects, Avascular necrosis, which is the death of bone tissue due to a lack of blood supply, is of particular interest, as there have been several recent reports [54] that have identified it as an adverse side-effect caused due to Prednisone. However, there is still no action taken by the health authorities in this regard.

Singulair is a drug commonly used in the treatment of asthma, especially in children. One of the most common novel negative side-effect identified from the messages in the forum was seizures. Apart from this, a less common but more adverse reaction that was identified from the forums was that of suicide. In March 2008, FDA had issued an early communication about investigation of a causal relationship between the drug Singulair and suicides [55]. Following the investigation, in August 2009, the FDA required an update to the Precautions section of the drug label to include information about neuropsychiatric events reported in patients using this drug [56].

Byetta is a drug used in the treatment of type 2 diabetes. The most common side-effects of this drug include nausea, vomiting, diarrhea and dizziness. The number of messages that were available in the message forum relating to this drug were only 10 and only 3 of them were identified to contain adverse side-effects. However, all 3 of them identified cancer as one of the side-effects. In 2009, FDA had issued a safety update for Healthcare Professionals [57] regarding Byetta warning them about the risks of the drug causing acute pancreatitis and altered kidney function. The update required the drug manufacturer to conduct further post-marketing studies to identify the incidence and risk factors for the adverse reactions, in addition to exploring the potential signal for a serious risk of thyroid and pancreatic cancer.

The above instances provide the necessary examples of why such automated mining systems would be valuable in identifying unreported adverse reactions and display the capability of our system in identifying such novel adverse side-effects. The main advantage of the proposed approach is the volume and timeliness of the discovered information. That is, the capability of collecting very large amount of up-to-date information at very low cost. With the source of the data being the online healthcare forums, this approach leverages all the benefits of ‘Big Data’. The online forums which act as a source of ‘Big Data’ are able to provide extremely high volume of raw data that can be used to extract information – discover adverse drug reactions in our approach. With its high volume and diversity, it is able to cover a large number of drugs which are usually not possible to cover in case of clinical trials. While collecting similar information through clinical trials can be very expensive, crawling of data from online forums is almost free, with most of the data being publicly available without any access restrictions when compared to other Literary or Clinical sources. Also with the users constantly providing feedback on the forums, we are able to provide the most up-to-date information on the side-effects of drugs.

### Limitations

As with all the benefits leveraged from the ‘Big Data’ source, this approach also inherits some of its drawbacks. One of the major issue with user generated data from online healthcare forums is the amount of noise that could be present in such forum messages. Majority of the members of such forums are average users who don’t necessarily have any medical background, hence, they may provide inaccurate or exaggerated information when it comes to drug side-effects. Using such a source for mining of ADR data may potentially provide false positives. The size of data helps mitigating this problem – repeatedly reported side effects are more likely to be true positives. Also the reports might be biased, as users tend to not make forum posts when there are no side-effects observed on consumption of a drug. Therefore, we present the mined information as early indicators of potential ADRs, and these reports have to be further investigated through rigorous medical and clinical procedures by health authorities to confirm if the drugs involved indeed cause the reported adverse reactions.

### Future work

The results of the HMM classification are promising to explore further options for improving the performance. Being able to distinguish between symptoms and side-effects would help in reducing the number of False Positives and maintaining a list of symptoms for which a drug is prescribed might help in this regard. The HMM classifier could also be expanded to extract other health related data like drug-dosage, disease-treatment relationships from the online forums. The extracted data can then be mapped on to an ontology which can be queried to obtain more accurate and novel information.

## Conclusions

In this paper we have presented a novel Hidden Markov Model based text mining system that is capable of extracting adverse reactions of drugs based on content available from online healthcare forums. We have shown that the information extracted from this system matches published information available in Drug Package Inserts. In addition we have also been able to identify some novel adverse side-effect information that can act as early indicators for health authorities to help in their efforts towards Pharmacovigilance. The results are encouraging to pursue further enhancements to this approach.
